# Mating Competitiveness of Irradiated *Lobesia botrana* (Lepidoptera: Tortricidae) in Male-Only and Both Sex Release Strategies under Laboratory Cage Conditions

**DOI:** 10.3390/insects14010018

**Published:** 2022-12-23

**Authors:** George Saour, Ali Hashem, Iyad Jassem

**Affiliations:** Atomic Energy Commission of Syria, Damascus P.O. Box 6091, Syria

**Keywords:** European grapevine moth, sterile insect technique, inherited sterility, moth both sex release

## Abstract

**Simple Summary:**

The sterile insect technique/inherited sterility (SIT/IS) has been successfully implemented to suppress or eradicate pestiferous species of Lepidoptera. The SIT/IS involves the release of mass-reared and gamma-irradiated moths (partially sterile males and fully sterile females) to achieve matings with wild moths. The success of any SIT/IS project depends, *inter alia*, on the overflooding ratio of released sterile moths to wild moths and the efficiency of male-only versus bi-sex releases of irradiated moths. The current laboratory study aims to understand the efficacy of overflooding ratios and the use of male-only releases compared with bi-sex releases for SIT/IS against the European grapevine moth, *Lobesia botrana* (Denis and Schiffermüller).

**Abstract:**

This laboratory study explored the concept of whether irradiated male-only releases are more or equally efficient as releases of both irradiated males and females in the context of using the sterile insect technique/inherited sterility (SIT/IS) for the management of the European grapevine moth *Lobesia botrana*. The current study examined the mating competitiveness of 150-Gy-treated *L. botrana* male and female moths or 150-Gy-treated male moths only, with untreated moths in laboratory cages. Our results showed that the release of both sexes significantly increased the competitiveness value (*C*) and the biological efficiency index (*BE*) as compared with male-only release, and this was independent of the male to untreated male ratio. Moreover, a single release of 150-Gy-treated and untreated males and females at a 1:1:10:10 ratio (untreated male:untreated female: treated male:treated female) significantly reduced egg hatch, and the number of first-generation offspring (F_1_) was small. The emergence of F_2_-moths per untreated F_1_ male and female moth was low, but these undesired fertile moths should be eliminated in order to achieve effective control. The results presented herein provide useful information on the impact of 150-Gy-treated male-only, versus releases of both treated males and females on untreated moths, which is essential to managing *L. botrana* populations with SIT/IS.

## 1. Introduction

Grape (*Vitis vinifera* L.) is an important cultural, economic, and ecological crop worldwide, with the largest acreage and the highest economic value among fruit crops [1]. According to the Food and Agriculture Organization of the United Nations, grapes are cultivated on 75,866 square kilometers worldwide [2]. The European grapevine moth *Lobesia botrana* (Denis and Schiffermüller; Lepidoptera: Tortricidae) is among the most destructive insect pests of grapes, threatening vineyards in southern Europe, the Middle East, and South America [3]. Populations of *L*. *botrana* are to date mainly controlled using broad-spectrum insecticides, biological control agents, and mating disruption [4,5,6]. Although all of these techniques have achieved effective control of this pest [7], they also have some limitations, i.e., development of insecticide resistance in the moths, contamination of the environment, health risks of pesticides, consumer choices for organic foods, and the need for maintaining adequate pheromone coverage in orchards [8,9]. The sterile insect technique/inherited sterility (SIT/IS) as part of an area-wide integrated pest management (AW-IPM) approach has proven to be a robust and effective control tactic for the management of lepidopteran pests. In the SIT/IS technique, a sub-sterilizing gamma dose is used for the male moths that are destined for release to suppress the reproduction of a target species. Good examples are the suppression of the codling moth *Cydia pomonella* L. (Lepidoptera: Tortricidae) in the Okanagan Valley of British Columbia, Canada, the eradication of the pink bollworm *Pectinophora gossypiella* (Saunders) (Lepidoptera: Gelechiidae) in the southern USA and northern Mexico, and the suppression of the false codling moth *Thaumatotibia leucotreta* (Meyrick) (Lepidoptera: Tortricidae) in South Africa. Therefore, this study is part of an assessment to examine whether the SIT/IS could be integrated in an AW-IPM approach for the suppression of populations of *L*. *botrana* [10,11,12].

Current programs using SIT/IS against lepidopteran pests rely on mass-rearing and releases of both male and female moths that have been treated with gamma irradiation [13]. There are currently no decisive data available to conclude whether the release of irradiated male moths only would provide significant economic gains (decreasing operational costs) or increase the efficiency of the SIT/IS in the field as compared with the release of both irradiated genders [14]. Genetic sexing strains that allowed the mass production and release of only males have been developed for the Mediterranean fruit fly, *Ceratitis capitata* (Wiedemann) (Diptera: Tephritidae), and this has significantly increased the efficiency of the technique in the field and greatly reduced programme costs [15]. In contrast, data from field-cage experiments with the cactus moth *Cactoblastis cactorum* Berg (Lepidoptera: Pyralidae) and *C*. *pomonella* suggest that the presence of sterile females might have a positive impact on population suppression [16,17]. Van Steenwyk et al. [18] have shown that sterilized females of *P*. *gossypiella* were equal to or more competitive than normal females. Recently, Sengupta et al. [19] reported that the simultaneous release of sub-sterile male and sterile female moths of *Spodoptera litura* (Fabr.) (Lepidoptera: Noctuidae) using the IS technique might improve its efficacy, leading to more effective pest suppression. Moreover, Ikegawa et al. [20] found in a mathematical model that the bi-sex release of sterile males and females can be a compatible measure to suppress and eventually eradicate wild pest populations. Thus, there is a need to obtain conclusive data from tests that would compare the efficiency of male-only versus both sex releases for the control of lepidopteran pests [21].

Efforts to develop efficient genetic sexing strains in Lepidoptera have been successful in two species: the silkworm *Bombyx mori* L. (Lepidoptera: Bombycidae) and the Mediterranean flour moth *Ephestia kuehniella* (Zeller) (Lepidoptera: Pyralidae) [22,23]. However, Marec et al. [24] pointed out that the developed system (a balanced lethal strain) is not practical for lepidopteran SIT/IS programs as it requires the maintenance of two colonies, the manual separation of sexes in both colonies, and crosses that produce male-only progeny. In addition, it requires the regular verification of the genetic structure of the balanced lethal strain. However, it is worth mentioning that there has been progress on transgenic/CRISPR to create male-only stains in several economically important Lepidoptera species [25]. While waiting for the production of transgenic *L*. *botrana* stain in any current program using the SIT/IS approach against *L*. *botrana* [12], the irradiated male and female moths are released. 

The possibility of applying SIT/IS against *L*. *botrana* populations in the field has been previously reviewed, and it was found that 150-Gy-treated females were fully sterile when mated with untreated male moths [10,11]. However, to the best of our knowledge, no study has been conducted to examine the effects of releasing 150-Gy-treated *L*. *botrana* female moths on the mating competitiveness of 150-Gy-treated male moths and consequently on the suppression of *L*. *botrana* populations. In fact, if treated male and female moths are released together, the treated males may court the sterile females and consequently not seek out the wild females as effectively as if they had been released without sterile females. This interference effect of sterile females on treated males could have a significant impact on male mating effectiveness. On the positive side, sterile female moths could attract wild males and “distract” them from mating with wild females. Moreover, a synergistic benefit has been demonstrated where sterile eggs laid by irradiated females have been shown to enhance the numerical response of egg parasitoids [26,27].

The current laboratory study aimed to understand the efficacy of *L*. *botrana* male-only release compared with bi-sex release. This study also aimed to determine the effects of male: female sex ratios on the competitiveness of treated male and female moths on the suppression of *L*. *botrana* populations. Noting that competitiveness can be assessed in different ways [28], in the current study, the Fried test (*C* value) [29] was used to calculate mating competitiveness. Furthermore, a follow-up study in large cage experiments under laboratory conditions was performed to determine the effects of a single release of 150-Gy-treated and untreated moths on *L*. *botrana* F_1_ and F_2_ fertility. 

## 2. Material and Methods

Moths used in the experiments were derived from a colony maintained in the laboratory for several years. The colony was renewed (up to 50%) each year with field collected *L*. *botrana* larvae from green (unripe) grapes. The larvae were reared on a semi-artificial diet as described by Thiéry and Moreau [30] and Saour [11]. All insect stages were kept at a constant temperature of 25 ± 1 °C and RH of 70 ± 5%, and a photoperiod of 16:8 h (light:dark) + 1 h of dusk. 

### 2.1. Moth Irradiation

Newly emerged adult male and female *L*. *botrana* moths (<18-h old) were irradiated (at room temperature ≈ 23 °C) with a dose of 150 Gy in a Cobalt-60 gamma irradiator (Issledovatel Gamma Irradiator, Techsnabexport Co., Ltd., Moscow, Russia) at a dose rate of 9.8 Gy/min. Moths were placed individually into commercial grade small transparent plastic test tubes (12 × 75 mm) prior to the irradiation treatment. The dose uniformity ratio (max:min of the received dose) was about 1.14 (measured by the Fricke test), and the absorbed dose was calibrated using the conventional Fricke dosimetry.

### 2.2. Experiment 1 and 2: Effect of 150-Gy-Treated Male and Female Moths on Percentage Egg Hatch in Laboratory Cages at Different Sex Ratios

Wooden-framed, rectangular oviposition cages (65 × 45 × 45 cm) were used that had one side that could be opened and closed. The cages were covered with stainless steel woven mesh (0.5 mm), except for one side that was made of a removable transparent nylon sheet as a smooth oviposition support. In order to prevent gravid female moths from laying eggs on the cage’s wooden frame and induce them to lay eggs on the oviposition support, the bottom and wooden frame inside the cage were covered with commercial sandpaper sheets (p 180) as a rough surface material. The moths were provided with a 5% sucrose solution *ad libitum* through a soaked cotton wick. The oviposition cages were maintained under the abovementioned laboratory rearing conditions. 

In a first experiment, males irradiated with 150 Gy were confined with untreated males and female moths at the following ratios: 1:1:1, 1:1:5, and 1:1:10 (untreated ♂:untreated ♀:150-Gy-treated ♂). Every ratio was replicated three times with a single release of 30, 75, and 90 treated males for 1:1, 1:5 and 1:10 untreated to treated male ratios, respectively.

In a second experiment, male and female moths that had been irradiated with 150 Gy were confined with untreated male and female moths at the following ratios: 1:1:1:1, 1:1:5:5, and 1:1:10:10 (untreated ♂:untreated ♀:150-Gy-treated ♂:150-Gy-treated ♀). Ratios of 1:1:0:0, 0:1:1:0, 1:0:0:1, and 0:0:1:1 were used as control groups. Every ratio was replicated three times with a single release of 60, 100, and 120 treated male and female moths for 1:1:1:1, 1:1:5:5, and 1:1:10:10 ratios, respectively. 

Every 2 days, the oviposition sheet (a nylon sheet) was removed and replaced with a new sheet, and the deposited eggs were counted and incubated in closed transparent plastic boxes at 25 °C for 1 week before determining the percentage of egg hatch. The total number of eggs laid/females at each ratio and the total number of eggs hatched at each ratio/total number of eggs laid at each ratio) were calculated.

The percentage of expected egg hatch and male mating competitiveness (*C* value) in the case of releasing only 150-Gy-treated male moths was estimated by using Fried’s formula based on egg hatch data as follows [29]:

*Expected egg hatch* = *Hn* × [*N*/(*N* + *T*)] + *Ht* × [*T*/(*N* + *T*)] and *C* = (*Hn* − *He*/*He* − *Ht*) × *N*/*T* (1)

where *Hn* is the observed % egg hatch when untreated male and female moths were kept together; *Ht* is the observed % egg hatch when treated male and untreated female moths were kept together; *He* is the observed % egg hatch when treated males were combined with untreated male and female moths; *N* is the number of untreated male moths; and *T* is the number of treated male moths.

The percentage of expected egg hatch and *C* value in the case of releasing 150-Gy-treated male and female moths were calculated by using modified formulae from Fried’s test as follows:

*Expected egg hatch* = *Hnn* × [*NN*1/(*NN*1 + *TT*1)] + *Htt* × [*TT*1(*NN*1 + *TT*1)] *C* = 1 − [(*Hnn* − *Htt*) × *Htt* − 1/(*Hnn* − *He*_1_) × (*He*_1_ + 1) − 1] (2)

where *Hnn* is the observed % egg hatch when untreated male and female moths were kept together; *Htt* is the observed % egg hatch when treated male and female moths were kept together; *NN*1 is the number of untreated male and female moths; *TT*1 is the number of treated male and female moths; and *He*_1_ is the observed % egg hatch when treated male and female moths were combined with untreated male and female moths.

Moreover, the biological efficiency index (BE) [31,32] was used (as an additional calculation) to evaluate the effectiveness of using either 150-Gy-treated male moths or both 150-Gy-treated sexes. The following formula was used:

*BE* = 1 − (*R*/*T*) × 100%

where *R* is the total number of eggs hatched when treated moths (of one-sex or both-sex) were paired with untreated ones at each ratio; *T* is the total number of eggs hatched in the unirradiated control group (untreated male × untreated female).

### 2.3. Experiment 3: Effect of the Presence or Absence of 150-Gy-Treated Females on Egg Hatching

Metal commercially made oviposition cages of 1.1 m^3^ (1.0 m long × 1.0 m wide and 1.1 m high) were used for this experiment and consisted of a metal frame covered with a stainless steel woven mesh (0.5 mm). Similar to the wooden cage, one side of the cage had a rectangular opening of 40 × 80 cm and was covered with a transparent nylon sheet as an oviposition support. A hole of 10 cm diameter was also available on one side of the cage (in the middle of the left side of the clear nylon sheet) to be able to handle the moths inside the cage. The hole was covered with a piece of muslin cloth (a sleeve) fastened with a rubber band.

In this experiment, 150-Gy-treated males (*n* = 100) were confined with untreated male and female moths at a mating ratio of 1:1:10:0, or 150-Gy-treated males and females (*n* = 200) were confined with untreated male and female moths at a mating ratio of 1:1:10:10 (untreated ♂:untreated ♀:150-Gy-treated ♂:150-Gy-treated ♀). Mating ratios of 1:1:0:0, 0:1:1:0, 1:0:0:1, and 0:0:1:1 were used as control groups. As in previous experiments, the eggs were collected (oviposition sheet) every 2 days, counted and incubated for 1 week before determining the percentage of egg hatch. The cages were placed in a controlled room and maintained at the rearing conditions outlined above. The percentages of expected egg hatching, *C*, and *BE* were estimated by using the modified Formula (2). The experiment was replicated three times at each mating ratio.

### 2.4. Follow-Up Study on F_1_ and F_2_ Fertility

The experiment was designed to assess radiation-induced inherited effects on F_1_ and F_2_ progeny. 

#### 2.4.1. Parental Generation

Newly emerged (<18-h old) males and females were treated with 150-Gy and confined with untreated male and female moths at the ratio of 1:1:10:10 (untreated ♂:untreated ♀:150-Gy-treated ♂:150-Gy-treated ♀) in the 1.1 m^3^ oviposition cage (a single release of 10:10:100:100 treated and untreated male and female moths). Every 2 days, the oviposited eggs were collected (an oviposition sheet), counted, and incubated in closed, transparent plastic boxes for hatching. The newly hatched larvae were individually placed on small pieces of the semi-artificial diet using a fine camel’s-hair brush. Larvae were checked daily until pupation, and the sex ratio of the emerging moths was determined. 

#### 2.4.2. Fertility of F_1_

The newly emerged F_1_ male and female moths were collected and singly paired (in 9-cm-diameter and 1-cm-high transparent plastic Petri dishes) with virgin, untreated colony adults of the opposite sex. Each Petri dish was provided *ad libitum* with a 5% sucrose solution as a food source through a soaked cotton wick. The inner Petri dish surface served as the oviposition substrate. The females were kept for oviposition until death. The oviposited eggs from each pair were collected, counted, and left to determine the percentage of egg hatch. Based on previous findings, the fertility of *L*. *botrana* will vary from 20–25% for F_1_ moths from 150-Gy-treated male parents to 75–85% for untreated moths. Thus, each pair was considered partially sterile or fully fertile depending on the assessment of their percentage egg hatch. The experiment was replicated twice.

### 2.5. Statistical Analysis

In order to identify significant differences between the percentages in all mating competitiveness experiments (i.e., between observed and expected egg hatch), a normal approximation test (Z) at a 95% confidence interval (*p* ≤ 0.05) was performed where necessary for comparing two percentages using the Stat-View program [33].

## 3. Results

### 3.1. Experiments 1 and 2

In the first experiment, only 150-Gy-treated males were released, and a significant difference (i.e., Z = 4.2 for the 1:1:1 ratio; *p* ≤ 0.05) was found between the observed and expected egg hatch except when the 1:1:10 ratio was used. The *C* value augmented with the increased ratio of sterile males, i.e., from 0.19 with a ratio of 1:1:1, to 0.76 with a ratio of 1:1:10 (Table 1). Similarly, the *BE* improved by increasing the ratio of treated to untreated males, i.e., from 0.74 to 83.2, with ratios of 1:1:1 and 1:1:10, respectively.

In the second experiment, 150-Gy-treated males and females were released, and no significant difference was found between the percentage of expected and observed egg hatch at each used ratio (i.e., Z = 0.78 for the 1:1:1:1 ratio; *p* ˃ 0.05). However, fertility was reduced with an increased ratio of treated male and female moths (e.g., 35.4 and 7.9% for ratios of 1:1:1:1 and 1:1:10:10, respectively). Moreover, both *C* and *BE* values were increased when male and female moths were released at the 1:1:10:10 ratio as compared with the two other tested ratios (e.g., *C* values were 0.15 and 0.87 for ratios of 1:1:1:1 and 1:1:10:10, respectively) (Table 1). 

More importantly, Table 1 shows that the *C* and *BE* values were higher when both sexes were released as compared with when only 150-Gy-treated males were considered (except for the *C* at a 1:1:1 ratio). For example, the *C* values were 0.40 and 0.74 and the *BE* values were 62.7 and 74.0, with ratios of 1:1:5 and 1:1:5:5, respectively. 

### 3.2. Experiment 3

Releasing 150-Gy-treated males only with untreated males and females at the ratio of 1:1:10:0 resulted in a *C* value of 0.64 and a *BE* value of 78.7. While releasing treated males and treated females (ratio of 1:1:10:10), the *C* value increased significantly to 0.90 (Z = 4.56; *p* ≤ 0.05) and the *BE* index increased to 95.6 (Table 2). Moreover, no difference was found between the percentage observed and expected egg hatch (i.e., Z = 0.05 for a 1:1:10:10 ratio; *p* ˃ 0.05) at the used dose and mating ratios (Table 2). 

### 3.3. Follow-Up Study on F_1_ and F_2_ Fertility

Data on the fertility of F_1_ male and female moths resulting from crosses at the mating ratio of 1:1:10:10 are provided in Table 3. Out of 1873 eggs oviposited, 101 eggs hatched (fertility of 5.4%), indicating that egg hatch resulted from the crosses between treated male × untreated female or untreated male × untreated female, whereas the majority of laid eggs (1722 eggs, 94.6%) are from treated females since any crosses with these females with will give zero percent egg hatch. For the F_2_ fertility follow-up study, 90 pairs were formed, based on the assessment of their observed fertility, the majority of which resulted from F_1_ males or females from treated male parent × untreated male or female moths. However, a small number of F_2_ adults (90/5, 5.5%) emerged from eggs of untreated females that had mated with untreated male moths.

## 4. Discussion

In the present study, a first attempt was made to assess the effect of 150-Gy-treated *L*. *botrana* male-only releases as compared with releases of both treated males and treated females in oviposition cages under laboratory conditions.

A *C* value that is equal to one indicates that the irradiated male moths are equally competitive with untreated males [34,35,36], while a higher *BE* is an indication of a more effective release method [31]. *BE* values will be equal to 100 at 1:0:0:1 and 0:0:1:1 ratios because the fertility of 150-Gy-treated females will result in zero egg hatch if they mate with either treated or untreated males.

The decrease in fertility observed in experiment 1 was not sufficient when treated males were released at 1:1:1 and 1:1:5 ratios because the male moths used in our experiments were partially sterile. By increasing the ratio of treated males to untreated males to 10 to 1, the *C* and *BE* values increased. Similar results were reported when higher ratios of partially sterile male moths of the potato tuber moth, *Phthorimaea operculella* Zeller (Lepidoptera: Gelechiidae), were used [37].

The release of both treated male and female moths requires the availability of adequate mating indices to ascertain the relative importance of the competitiveness of each sex [28]. Therefore, Fried’s formulae (the percentage expected egg hatch and the *C* value) were modified to allow calculation of the effectiveness of both sex releases of treated male and female moths. Moreover, using the biological efficiency (BE) index in the current study is an appropriate index because eggs hatch only from untreated females (as treated females are completely sterile). Our results indicate that releasing both sexes reduced the observed egg hatch and improved *BE* values regardless of the mating ratio (Table 1). For example, comparing 1:1:5 with 1:1:5:5 ratios, the *C* and *BE* values increased by 45.9% and 16.2%, respectively, and this could solely be attributed to the presence of both fully sterile females and partially sterile males. A similar result has been reported when *P*. *operculella* male and female moths were released in 350 mL transparent plastic oviposition boxes [32].

The use of larger 1.1 m^3^ cages and higher numbers of adult moths (up to two hundred 150-Gy-treated and untreated adults) resulted in more precise and informative data in comparison with the data obtained from the experiments carried out in the wooden cages that used fewer treated and untreated moths. Releasing both treated male and female moths in the larger oviposition cage at the 1:1:10:10 ratio improved the *C* value and the *BE* index by 40.6 and 21.5%, respectively, as compared with the release of only treated male moths (1:1:10:0 ratio). The use of a larger oviposition cage increased the effectiveness of the bi-sex release versus releasing males only. Further studies in field cages and field conditions are needed to confirm our findings.

Our data are not in agreement with the results of earlier studies [38,39] reporting that the release of males only was more efficient than the release of both irradiated sexes of *C*. *pomonella* and *P*. *operculella*. However, it needs to be noted that the abovementioned studies were not specifically designed to evaluate factors and variables related to the competitiveness of irradiated females. It is likely that their findings could have been attributed to the deterioration of the female’s behaviour and physiology due to the application of the high irradiation doses of 400–450 Gy. In contrast, our results confirm Ikegawa et al.’s [20] data that sterile female moths can potentially contribute to more effective pest control using the SIT, particularly if sterile males have a lower sexual performance than wild males.

It is well documented that lower doses of radiation used to induce F_1_ sterility in lepidopteran species increase the quality and competitiveness of the released moths [40,41]. However, a limitation of a pest control program that has an SIT/IS component is the difficulty of distinguishing F_1_ progeny (resulting from the mating of a partially sterile released male with a wild female moth) from wild fertile moths. A cytological technique exists for several lepidopteran species to identify F_1_ males based on orcein and Giemsa staining of the sperm [40]. The method is, however, tedious and time-consuming. In our F_1_ follow-up study, the percentage of egg hatch was used as an indicator to distinguish between F_2_ progeny that resulted from untreated *L*. *botrana* females who had mated either with treated or untreated male moths.

The large cage study that followed the smaller cage studies suggests that a single release of treated and untreated male and female moths at a 1:1:10:10 overflooding ratio reduced egg hatch significantly and resulted in few F_2_ progeny. The F_2_ eggs from untreated F_1_ males/females × untreated males/females that possess the potential for inherited sterility represented about 95% of the formed pairs. Although the emergence of fertile F_2_-moths was low, the presence of these undesired moths needs to be avoided by increasing the ratio of treated male and female moths as compared with untreated moths.

The data of our study indicate that the efficiency of SIT/IS against the *L*. *botrana* population can be improved by releasing both 150-Gy-treated partially sterile males along with fully sterile female moths. The benefit of this release method might be perceived one generation after the release, when wild females mate with treated males. Given that the F_1_ progeny inherit the detrimental effects of exposing male moths to 150 Gy of γ-radiation (offspring with a high level of sterility), no egg hatch will occur when 150-Gy-treated females mate with wild male moths.

Even though this study is based on laboratory bioassays that do not reflect many conditions experienced upon release as well as other prevailing conditions that exist in vineyards, the encouraging findings should nevertheless enhance motivation for further investigations on the use of SIT/IS against *L*. *botrana* at an overflooding ratio of 1:1:10:10 in field cages and even open field experiments.

## 5. Conclusions

Over the past years, extensive research has provided valuable knowledge that paved the way for the use of the SIT/IS approach against lepidopteran pests. In any SIT/IS program to manage *L*. *botrana*, irradiated male and female moths are released together since the manual separation by sex of thousands of moths may not be feasible. The bisexual release, in laboratory oviposition cages, of irradiated *L*. *botrana* moths is more effective than male-only release. Moreover, an overflooding ratio of sterile:wild moths is needed to avoid the emergence of fertile F_2_- moths. Finally, carrying out the current experiments in semi-field or field cage conditions is essential in order to consolidate our findings. 

## Figures and Tables

**Table 1:** Percentages of observed and expected egg hatch, competitiveness value (*C*) and biological efficiency (*BE*) index when 150-Gy-treated *L*. *botrana* males and females were placed with untreated male and female moths in an oviposition cage (65 × 45 × 45 cm) at different mating ratios. (N = Untreated male; N1 = untreated female; T = 150-Gy-treated male; T1 = 150-Gy-treated female).

Mating Ratios	No. of Moths	Observed Egg Hatch (%)	Expected Egg Hatch (%)	Competitiveness Value (*C*)	Biological Efficiency Index (*BE*)
(Males only) N:N1:T					
1:1:0	30:30:0	79.9 (2967/3714)	-	-	-
0:1:1	0:30:30	40.3 (1415/3510)	-	-	52.3
1:1:1	30:30:30	73.6 (2945/4001) a	60.1 b	0.19	0.74
1:1:5	15:15:75	53.5 (1107/2070) b	46.2 b	0.40	62.7
1:1:10	9:9:90	44.9 (497/1107) a	43.8 a	0.76	83.2
(Males + Females)					
N:N1:T:T1					
1:1:0:0	30:30:0:0	80.9 (2767/3420)	-	-	-
0:1:1:0	0:30:30:0	39.1 (1323/3386)	-	-	52.2
1:0:0:1	30:0:0:30	0 (0/2340)	-	-	100
0:0:1:1	0:0:30:30	0 (0/2098)	-	-	100
1:1:1:1	30:30:30:30	35.4 (2067/5841) a	40.5 a	0.15	25.3
1:1:5:5	10:10:50:50	14.8 (720/4860) a	13.4 a	0.74	74.0
1:1:10:10	6:6:60:60	7.9 (386/4896) a	7.4 a	0.87	86.0

Each ratio was repeated three times. Numbers between parentheses are hatched eggs/total laid eggs at each ratio. Percentages in row followed by the same letter are not significantly different (*p* ≤ 0.05, *Z* test).

**Table 2:** Percentages of observed and expected egg hatch, competitiveness value, and biological efficiency index when 150-Gy-treated *L*. *botrana* males and females were placed with untreated male and female moths in a 1.1 m^3^ oviposition cage at 1:1:10:0 or 1:1:10:10 mating ratios. (N = untreated male; N_1_ = untreated female; T = 150-Gy-treated male; T_1_ = 150-Gy-treated female).

Mating Ratios N:N_1_:T:T_1_	No. of Moths	Observed Egg Hatch (%)	Expected Egg Hatch (%)	Competitiveness Value (*C*)	Biological Efficiency Index (*BE*)
1:1:0:0	50:50:0:0	81.0 (3965/4895)	-	-	-
0:1:1:0	0:50:50:0	38.2 (1664/4359)	-	-	57.2
1:0:0:1	50:0:0:50	0 (0/650)	-	-	100
0:0:1:1	0:0:50:50	0 (0/598)	-	-	100
1:1:10:0	10:10:100:0	44.0 (846/1924) A	41.7 A	0.64 b	78.7
1:1:10:10	10:10:100:100	5.8 (173/2982) A	7.4 A	0.90 a	95.6

The treatment was repeated three times. Numbers between parenthesis are hatched eggs/total laid eggs. Percentages in rows followed by the same uppercase letter are not significantly different (*p* ≤ 0.05, Z test); Percentages in columns followed by the same lowercase letter are not significantly different (*p* ≤ 0.05, Z test).

**Table 3:** Fertility of *L. botrana* F_2_ male and female moths resulting from 150 Gy-treated male mated to untreated female or untreated male mated with untreated female moths (parental generation) when released in a oviposition cage of 1.1 m^3^ at 1: 1: 10: 10 (untreated ♂: untreated ♀: 150 Gy-irradiated ♂: 150 Gy- irradiated ♀) mating ratio.

Generations	Crosses	Type of Eggs	No. of Laid Eggs	No. of Hatched Eggs	No. of Initial Pairs	No. of Partially Sterile Pairs	No. of Fertile Pairs	F_2_ Mean Egg Hatch (%)
Parental (P_1_)	150 Gy-treated ♂ X Untreated ♀	Partially sterile eggs	1873	-	-	-	-	-
	150 Gy-treated ♂ X 150 Gy-treated ♀	Fully sterile eggs		-	-	-	-	-
	Untreated ♂ X 150 Gy-treated ♀	Fully sterile eggs		-	-	-	-	-
	Untreated ♂ X Untreated ♀	Fertile eggs		-	-	-	-	-
F_1_	F_1_ ♂ from treated male parent X Untreated ♀	Partially sterile eggs	-	101	60	57	-	18.6 ± 4.9
	F_1_ ♀ from treated male parent X Untreated ♂	Partially sterile eggs	-		30	28	-	24.2 ± 3.9
	F_1_ ♂ from untreated parent X Untreated ♀	Fertile eggs	-		-	-	3	82.6 ± 2.3
	F_1_ ♀ from untreated parent X Untreated ♂	Fertile eggs	-		-	-	2	79.3 ± 2.9

The treatment was repeated twice, and 220 male and female moths were released at each treatment.

## Data Availability

Data are available upon request from the authors.
